# Physical Inactivity: A Modifiable Risk Factor for Morbidity and Mortality in Kidney Transplantation

**DOI:** 10.3390/jpm11090927

**Published:** 2021-09-18

**Authors:** Claudio Ponticelli, Evaldo Favi

**Affiliations:** 1 Nephrology, Ospedale Maggiore Policlinico, 20122 Milan, Italy; ponticelli.claudio@gmail.com; 2 Kidney Transplantation, Fondazione IRCCS Ca’ Granda Ospedale Maggiore Policlinico, 20122 Milan, Italy; 3 Department of Clinical Sciences and Community Health, University of Milan, 20122 Milan, Italy

**Keywords:** physical activity, sedentary behavior, exercise, sport, cardiovascular disease, frailty, quality of life, outcomes, kidney transplant, review

## Abstract

In patients with chronic kidney disease, sedentary behavior is widely recognized as a significant risk factor for cardiovascular disease, diabetes, obesity, osteoporosis, cancer, and depression. Nevertheless, the real impact of physical inactivity on the health of kidney transplant (KT) recipients remains uncertain. Over the last decade, there has been a renewed interest in exploring the effects of regular physical exercise on transplant-related outcomes. There is now mounting evidence that physical activity may reduce the burden of cardiovascular risk factors, preserve allograft function, minimize immunosuppression requirement, and ameliorate the quality of life of KT recipients. Many positive feedbacks can be detected in the early stages of the interventions and with a minimal exercise load. Despite these encouraging results, the perceived role of physical activity in the management of KT candidates and recipients is often underrated. The majority of trials on exercise training are small, relatively short, and focused on surrogate outcomes. While waiting for larger studies with longer follow-up, these statistical limitations should not discourage patients and doctors from initiating exercise and progressively increasing intensity and duration. This narrative review summarizes current knowledge about the deleterious effects of physical inactivity after KT. The benefits of regular physical exercise are also outlined.

## 1. Introduction

Among the many modifiable factors that can increase the risk of morbidity and mortality in kidney transplant (KT) recipients, physical inactivity is often neglected. This attitude is certainly questionable since a sedentary lifestyle has been widely recognized as a “silent killer” and a major health issue in the general population, particularly in more economically developed countries [1,2,3]. It is now well-known that physical inactivity is independently associated with a higher risk of cardiovascular disease (CVD), diabetes mellitus, obesity, osteoporosis, and breast and colonic cancer [4,5]. On the other hand, there is mounting evidence that regular physical activity can effectively prevent several chronic diseases, depression, and premature death [6,7].

The World Health Organization (WHO) has defined physical activity as any bodily movement produced by skeletal muscles that requires energy expenditure. The intensity of physical activity ranges from light (slow walking) to moderate (brisk walking, jogging, stair-climbing) to vigorous (fast running, sprinting, fast cycling). In order to reduce the burden on global health, the WHO has also recently provided guidelines on physical activity and sedentary behavior for children, adolescents, adults, and older adults. The guidelines for adults (namely, subjects between 18 and 64 years) recommended a minimum of 150–300 min of moderate-intensity aerobic physical activity, 75–150 min of vigorous-intensity aerobic physical activity, or an equivalent combination of both throughout the week to achieve substantial benefits. Adults should also carry out muscle-strengthening activities at moderate or greater intensity, with the involvement of all major muscle groups, at least twice a week. For older adults, the recommendations are similar. This group should perform varied multicomponent physical activity that emphasizes functional balance and strength training at moderate or greater intensity, three or more days a week [8].

To date, the real impact of physical inactivity on KT-related outcomes remains uncertain. In the present narrative review, we will summarize current knowledge about the possible deleterious effects of sedentary behavior in KT recipients. Potential benefits of physical activity will also be outlined.

## 2. Physical Activity before and after Kidney Transplantation

Current acceptance criteria for KT candidates are far more liberal than in the past. Even though there is still considerable variability in recipient-selection policies among centers worldwide, it is undeniable that many conditions previously recognized as an absolute contraindication to transplantation are now considered as a marginal issue. Progressively, we have witnessed the enlistment of more and more frail patients, often at extreme ages or with multiple comorbidities. One of the many consequences of such a shift of vision is that a number of potential transplant recipients, including children, exhibit low exercise capacity and are physically inactive [9,10]. The specific burden of chronic kidney disease (CKD) should also be taken into account. Indeed, elderly subjects waiting for a KT usually show inferior physical performances and are at higher risk of disability than age-matched individuals with other non-communicable chronic diseases [11]. Additionally, it has been observed that patients on regular dialysis undergo physical activity only nine days per month, with 43.9% not exercising at all [12]. Indeed, it seems that inactivity-related morbidity increases with the duration and type of renal replacement therapy (RRT). As such, it is plausible that preemptive KT recipients may achieve better physical performances than patients requiring prolonged dialysis before transplant. On the other hand, recent studies have demonstrated that physical aerobic exercise can exert various beneficial effects on many clinical outcomes, both in patients with CKD and under dialysis [13,14].

In KT recipients, the intertwining of dialysis vintage, comorbid conditions, psychosocial, and socioeconomic factors, as well as chronic exposure to immunosuppression, can all negatively affect physical activity. Calcineurin inhibitors cyclosporine and tacrolimus may induce myopathy and muscular atrophy through inhibition of calcineurin, which is a regulator of muscle mass [15]. Mammalian targeting of rapamycin (mTOR) inhibitors sirolimus and everolimus can also cause muscular atrophy by inhibiting the Akt/mTOR pathway that regulates skeletal muscle hypertrophy [16]. Steroid administration is associated with decreased protein synthesis and increased protein catabolism and may lead to muscle atrophy [17].

Even after a successful transplant, it is not uncommon that patients continue to feel as disabled as they were while on dialysis. Furthermore, in this specific group of patients, physical inactivity, defined as achieving less than 30 min of moderate-intensity exercise per week, is now recognized as a strong predictor of all-cause mortality and death with allograft function. A combination of semi-structured interviews and surveys provided two months after transplant by 88 renal allograft recipients has reported that 76% of the participants were sedentary, 13% exercised irregularly, and only 11% exercised regularly [18]. An Italian retrospective survey of KT recipients with at least a 10-year follow-up gathered information on physical activity from 6.055 patients. According to the results, 51.6% of the recipients were active whilst 48.4% were inactive. The lowest inclination to physical activity was observed in overweight and obese subjects, as well as in patients with longer dialysis vintage and older age at transplant. Noticeably, the propensity score showed that graft function was better preserved among active KT recipients [19].

Often underrated, further barriers to regular physical activity and adherence to exercise programs after transplant are the cost of fitness centers, inadequate exercise guidelines, medications side effects, feelings of total-body weakness, and the paucity of dedicated programs, as well as the need for expensive insurance coverage for participants. These issues should also be addressed to assist patients in managing their transplants [20,21]. Moreover, some patients are concerned that physical activity may interfere with the function of the allograft. In clinical practice, they often ask whether running, hiking, or gymnastics can put their transplant at risk. For all these reasons, many transplant recipients, in particular elderly subjects and children, remain sedentary. In an attempt to overcome some of these obstacles, various strategies have been proposed. For patients with end-stage kidney disease, the opportunity to offer nurse-led intra-dialytic or home-based supervised exercise programs appears particularly intriguing [22], while for transplant recipients, improved adherence to physical activity and increased walking capacity have been obtained using home-based exercise interventions with the aid of remotely-monitored wearable devices, financial incentives, or health-engagement questions [23].

## 3. Consequences of Physical Inactivity

Skeletal muscle is now recognized as an endocrine organ producing a panel of cytokines and peptides, also known as myokines, that can communicate with other organs, including adipose tissue [24]. Myokines production is strictly dependent on muscle contraction. As such, any muscle dysfunction may ultimately affect myokines production and potentially cause deleterious effects on multiple organs and systems. Sedentary behavior is associated with muscle wasting and visceral fat accumulation. The combination of physical inactivity and adiposity are in turn associated with elevated plasma levels of pro-inflammatory molecules, in particular interleukin-6 (IL-6) and serum c-reactive protein (CRP) [25,26]. At first glance, this observation may seem paradoxical since IL-6 and other pro-inflammatory cytokines are transiently secreted in large doses by several metabolically active tissues during exercise and muscular contraction [27]. However, it has been shown that muscle disuse may lead to IL-6 resistance with subsequently elevated levels of circulating IL-6. In this setting, the increase in plasma IL-6 concentration that accompanies obesity and physical inactivity may represent a compensatory mechanism, resulting in persistent low-grade systemic inflammation [28]. Chronically low-grade systemic inflammation has been linked to the development of many inflammation-related diseases [29].

A number of retrospective studies in KT settings have suggested that low levels of physical activity independently predict post-transplant outcomes, including mortality, particularly in obese and elderly patients [30,31,32]. In another retrospective analysis on 540 KT patients, low physical activity (properly defined using validated questionnaires) was strongly associated with an increased risk of cardiovascular and all-cause mortality [33]. Similar results were obtained in an elegant prospective study evaluating long-term (8 years) outcomes of a cohort of 507 adult KT recipients assessed using the Physical Activity Scale for the Elderly at the time of transplantation. According to this scale, participants were sorted into three groups (tertiles). The overall mortality rate was about 25%, and most of the deaths were recorded in patients with a functioning allograft. Interestingly, multivariable Cox regression analysis showed a statistically significant association between the physical activity score and both recipient and transplant survival [34]. Research performed on 4034 KT recipients has reported that lower levels of physical activity are associated with a higher prevalence of obesity, diabetes mellitus, and CVD [35]. Other major consequences of poor physical activity are the loss of muscle mass and frailty, an independent risk factor for post-transplant adverse events, especially in aged patients [36,37]. As a matter of fact, in older subjects, low physical activity level combined with excessive time spent in sedentary behavior (physical activity level < 150 min per week and sedentary behavior ≥ 540 min per day) has been associated with frailty, resulting in a frailty prevalence ratio of 2.83 [38]. Physical inactivity in KT recipients is associated not only with increased mortality, but also with fatigue [39,40], depression [41], and inferior quality of life (QoL) [42,43]. Lastly, in a KT candidate, frailty may reduce the chances to be admitted to, or remain active on, the transplant waiting list [44,45,46].

In summary, available data show that post-transplant lack of regular exercise may negatively impact QoL and cause severe complications. Proper evaluation of physical activity levels at the time of enlistment could represent a valuable tool for risk stratification and prediction of post-transplant patient survival.

**Figure 1:**
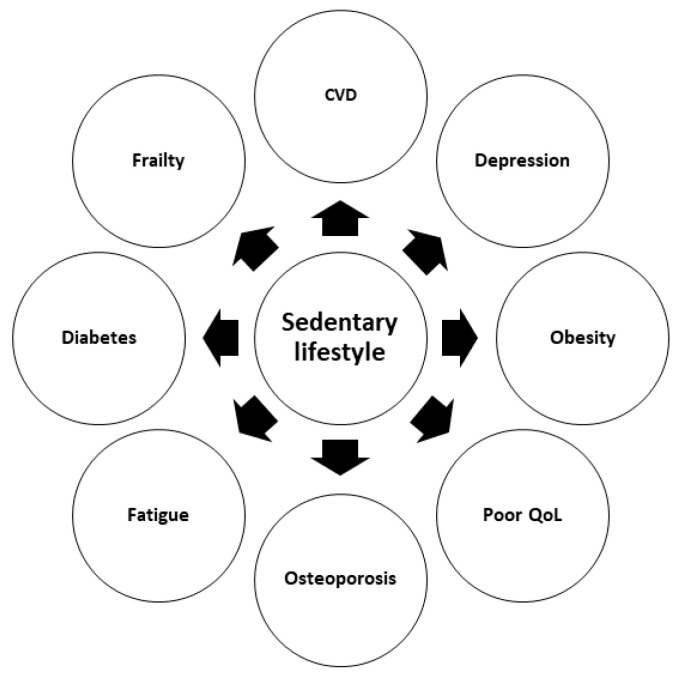
Negative effects of a sedentary lifestyle after kidney transplantation (CVD, cardiovascular disease; QoL, quality of life).

The negative effects of sedentary behavior on KT-related outcomes are summarized in Figure 1.

## 4. The Benefits of Physical Activity

CKD and long-term dialysis may lead to loss of muscle mass and, ultimately, to muscle atrophy [47]. After transplantation, these alterations cannot recover spontaneously nor rapidly. Adequate physical activity and regular exercise are fundamental and positively contribute to the rehabilitation process. The extent of the recovery is also strongly affected by the function of the transplanted kidney. In fact, recipients with normal allograft function are often highly motivated and can more easily restore their regular physical activity, thus improving their health and QoL.

As previously mentioned, exercise promotes a favorable anti-inflammatory milieu. A large study involving 4289 participants followed up for one year has shown that individuals reporting an increase in physical activity levels of at least 2.5 h per week exhibited lower CRP and IL-6 plasma concentrations than those remaining at their baseline physical activity level [48]. It is now accepted that sporadic exercise induces transient high-dose secretion of IL-6 and other pro-inflammatory cytokines by metabolically active tissues [49], while regular exercise training is associated with consistently low levels of systemic inflammatory markers [50] and reduction of visceral fat mass [51].

A central role in the regulation of skeletal muscle cells plasticity is played by peroxisome proliferator-activated receptor-γ coactivator-1α (PGC-1α). This protein is involved in the transcriptional regulation of genes participating in mitochondrial biogenesis, respiratory capacity, oxidative phosphorylation, and cellular events that control both muscle mass and function [52,53,54]. Physical exercise represents a powerful stimulus to the expression and signaling of PGC-1α [55,56]. Thus, maintaining optimal intra-cellular levels and activity of this transcriptional coactivator is crucial to protect the muscle from proteolysis, oxidative damage, and inflammation.

There is evidence that regular physical activity stimulates myocardial regeneration and ameliorates age-related loss of muscle mass and strength, a frequently overlooked modifiable risk factor for frailty and CVD [57,58,59,60,61]. A meta-analysis of 41 trials has compared exercise training with sham exercise or no exercise in 928 patients with CKD. The authors concluded that, overall, exercise interventions were correlated with improved aerobic capacity, muscular strength and motility, cardiovascular function, walking capacity, and health-related QoL. The vast majority of subjects included were on RRT and followed aerobic exercise programs [62]. According to a systematic review published in 2009, regular physical activity after KT leads to improved QoL and aerobic fitness, and it is negatively associated with body fat percentage [43]. A homocysteine-lowering randomized clinical trial on 3050 KT recipients has shown that, after a mean follow-up of four years, patients in the highest tertile of physical activity have significantly lower rates of cardiovascular events, cardiovascular-related mortality, and all-cause mortality than those in the lower tertiles [63]. These findings are in line with those reported by another group of researchers that used the SF-36 questionnaire to compare the health-related QoL between 168 sporty (namely, practicing different sports at low to moderate intensity 5 ± 4 h per week) and 97 non-sporty KT patients. The results showed that regular physical activity can significantly improve several SF-36 domains, such as General Health and Role—Emotional. Furthermore, they indicate that the benefits of sports activity go beyond the predictable impact on physical health to involve the psychological and social components of QoL [64]. Remarkably, many positive feedbacks can be already detected in the early stages of the interventions, and with a minimal amount of exercise load, in every age group.

Physical exercise may also reduce arterial stiffness. The most widely used parameter for arterial stiffness is pulse wave velocity (PWV), which measures the speed of arterial pressure waves along the aorta and large arterial vessels. It is usually calculated by dividing distance with pressure wave transit time at the two points of recording arteries [65]. In an interesting study, 46 KT recipients were assigned to aerobic training (*n* = 13), resistance training (*n* = 13), or usual care (*n* = 20), and had their PWV and peak oxygen uptake (VO2peak, the maximal rate of oxygen consumption measured during exercise of increasing intensity) assessed after 12 weeks of follow-up. Analyses of covariance, adjusted for baseline values, age, and dialysis vintage, revealed a statistically significant difference in mean PWV between patients allocated to aerobic or resistance training and those on usual care, with no intervention-related adverse events, overall cardiovascular events, or hospitalizations [66]. Given the results obtained in the short term, the authors extended the follow-up of the study to nine months. They were able to confirm a statistically significant difference in mean PWV between the resistance exercise and the usual care group, as well as a statistically significant difference in mean VO2 peak between the aerobic arm and the usual care one. Albeit small, this study strongly suggests that the improvements in arterial stiffness achieved with a short exercise intervention may last for several months [67].

Other less predictable benefits may arise from regular physical activity after KT. A small trial with 20 participants has shown that in a 12-month course of 3 days per week, resistance or aerobic training may lead to a significant increase in mean estimated glomerular filtration rate (+7.8 ± 3.0 mL/min/1.73 m^2^ per year; *p* = 0.02) in comparison to no intervention [68]. In another study, regular exercise was associated with a reduced incidence of new-onset diabetes mellitus after transplantation [69].

Some data also support the hypothesis that physical activity may have a positive impact on inflammation and immunity. Indeed, it has been observed that exercise can downregulate pro-inflammatory cytokines, reduce the expression of the adhesion molecules CD80 and CD86, and increase the number of T regulatory cells [70]. Königsrainer et al. have demonstrated that exhaustive physical exercise can cause a different pattern of gene expression in transplant recipients compared with healthy athletes, thus speculating that physical exercise might potentially reduce the need for post-transplant immunosuppressive medications [71].

A systematic review and meta-analysis of studies on exercise training in solid organ transplant recipients has concluded that available trials are small-sized, of relatively short duration, and focused on surrogate outcomes. Accordingly, exercise training remains a promising but unproven intervention for improving cardiovascular outcomes of solid organ transplant recipients [72]. Waiting for larger studies with longer follow-ups, these statistical limitations should not discourage patients and doctors from initiating exercise and progressively increasing intensity and duration.

In summary, current data suggest that physical activity can lead to better patient survival parameters. Nevertheless, not all the studies supporting the positive effects of physical activity are consistent with “causality” criteria (i.e., chronology and randomized interventional trials). Indeed, it might also be the case that patients with better survival are those able to perform physical activity.

**Figure 2:**
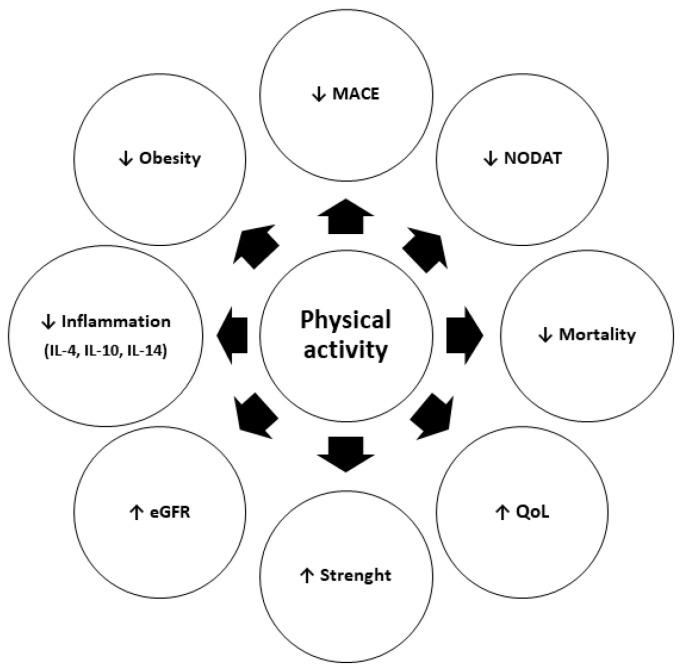
Positive effects of regular physical activity after kidney transplantation (MACE, major acute cardiovascular event; NODAT, new-onset diabetes after transplant; QoL, quality of life; eGFR, estimated glomerular filtration rate; ↑, increased; ↓, decreased).

The positive effects of regular physical activity on KT-related outcomes are summarized in Figure 2.

## 5. Physical Exercise and Sport after Transplantation

Since frailty and poor physical activity are associated with adverse outcomes after KT, physical performing tests, including the six-minute walk test and the sit-to-stand test, should be included in the evaluation of transplant candidates [73]. Furthermore, a rehabilitation program before surgery may be designed in frail candidates to prevent poor post-transplant outcomes. In a small pilot study, a pre-habilitation program consisted of weekly physical therapy sessions conducted at an outpatient clinic and at home. After two months of follow-up, enrolled subjects managed to improve their physical activity by 64% based on accelerometry. Five pre-habilitation participants received a KT during the study. Remarkably, their length of stay was shorter than in age-, sex-, and race-matched controls [74].

Fitness counseling should be offered to both patients on the transplant waiting list and KT recipients as a fundamental part of their routine medical care and follow-up programs. When promoting specific intervention strategies, healthcare professionals should always bear in mind that physical exercise may entail two major types of risk: osteo-skeletal and cardiovascular, depending on the physical activity and the underlying disease. There is a lack of formal recommendations on specific sports restrictions. The available literature is also extremely limited [75]. Even though there are examples of transplanted patients playing in professional soccer or rugby teams, it sounds reasonable to avoid full-contact impact sports that might result in injury to the area of the body where the kidney has been implanted. Prophylactic use of specifically designed padded sports clothing that protects the lower abdomen may be considered when practicing low- to moderate-risk activities. The burden of chronic allograft insufficiency and long-term immunosuppressive therapy should also be considered. Nonetheless, it seems appropriate to underline the fact that the perceived danger associated with sports and most concerns on physical activity-related safety issues after KT are often exaggerated. A systematic review of exercise training programs in KT patients has recently examined 24 studies with a cumulative population of 654 recipients and 536 controls. Included subjects were tested with an aerobic exercise program, a resistance exercise program, or a combination of both. Each intervention consisted of 20–60 min sessions, repeated 2–3 times per week for 5.5 months (median duration). The vast majority of the trials analyzed showed that all the interventions considered led to a significant increase in cardiorespiratory fitness and maximal heart rate and were associated with a substantial amelioration of muscle performance and strength. A trend towards weight loss in overweight or obese patients with stable allograft function, as well as an improvement in several aspects of QoL, were also noted. Remarkably, there was no harm in exercising or performing any physical activity [76]. These data show that regular exercise improves physical fitness and health-related QoL. It can also reduce overweight with a favorable effect on the cardiovascular system.

In clinical practice, particular attention should be paid to patients with specific comorbidities such as CVD, osteo-skeletal disorders, severe hypertension, or diabetes mellitus. Many physicians remain reluctant to prescribe formal exercise training programs to recipients with documented cardiovascular risk factors, but they only represent an absolute contraindication to physical activity in a small percentage of high-risk individuals [77]. Therefore, aerobic exercise is recommended in all KT recipients, and protocols consisting of moderate-intensity training exercises should be preferred [78]. These programs include outdoor activities such as walking, swimming, running, or cycling. However, aerobic exercises can also be efficiently performed indoors. High-Intensity Interval Training (HIIT) is a cardiovascular exercise strategy, alternating short periods of intense anaerobic exercise with less-intense recovery periods until complete exhaustion. This training can be performed using a cycling or a rowing ergometer, running, climbing stairs, or uphill walking. An example of a HIIT session that requires no equipment consists of a warm-up based on light jogging followed by five intervals of two minutes of near-maximal running. HIIT has been demonstrated to greatly improve the physical capacity of both healthy individuals and patients suffering from coronary artery disease or heart failure [79,80]. Although a recent study has shown that HIIT is a safe and effective exercise method in de novo heart transplant recipients [81], it has never been used in KT settings.

Today, a growing number of transplant recipients are dedicating themselves to sports and exercise training programs as a part of their constant effort to preserve and improve their health and well-being after transplantation. Several official competitions have been organized on a national and international scale. These events have the value of providing rare and important opportunities to build social relationships with other transplant recipients, as well as to widely promote physical activities and healthy lifestyles.

## 6. Conclusions

There is now emerging evidence that a sedentary lifestyle can be associated with an increased risk of cardiovascular events, a major cause of morbidity and mortality after KT [82,83,84,85]. On the other hand, regular exercise and physical activity may reduce the burden of cardiovascular risk factors and improve the perceived health status and QoL. We feel that, in everyday clinical practice, the physicians involved in the care of transplant recipients should pay particular attention to the lifestyle of their patients and, in the absence of formal contraindications, they should recommend regular physical activity. Focusing only on traditional recipient characteristics or transplant-related outcome measures may actually limit the beneficial effects of our day-by-day intervention strategies and may represent a missed opportunity for improvement. As recently stated by the World Kidney Day Steering Committee in the campaign “Living Well with Kidney Disease”, a disease-centered approach like the one we have been following for many years may be inadequate and may not reflect real patients’ priorities, needs, and values [86]. After prolonged periods of dietary restrictions, forced physical inactivity, and dialysis-related limitations in their recreational activities and job opportunities, it is reasonable to expect that those suffering from CKD, particularly after a successful transplant, want to regain their normality and sense of control over their health and well-being. In this regard, physical activity can certainly serve as a catalyst and help our patients take an important step toward a renewed self-centered existence.

## Data Availability

Data sharing not applicable. No new data were created or analyzed in this study.
